# Supplementary material for: Hepatic Sam68 Regulates Systemic Glucose Homeostasis and Insulin Sensitivity
Source: Int J Mol Sci. 2022 Sep 29;23(19):11469. doi: 10.3390/ijms231911469 (PMC9569775; doi:10.3390/ijms231911469)
Supplement: Supplementary file 1 [file ijms-23-11469-s001.zip › ijms-1860477-supplementary.pdf]

Hepatic Sam68 regulates glucose homeostasis and insulin sensitivity

Aijun Qiao, Wenxia Ma, Ying Jiang, Chaoshan Han, Baolong Yan, Junlan Zhou and Gangjian Qin

Supplementary Figures

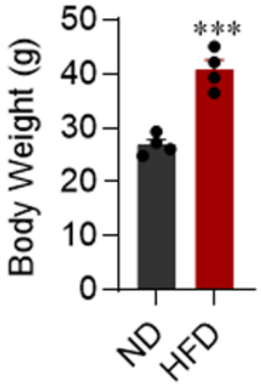

Figure S1. Body weight of C57BL/6J male mice fed on normal diet (ND) and high fat diet (HFD) for 12 weeks. n=4 per group. ***p < 0.001. Unpaired two-tailed Student's *t* test.

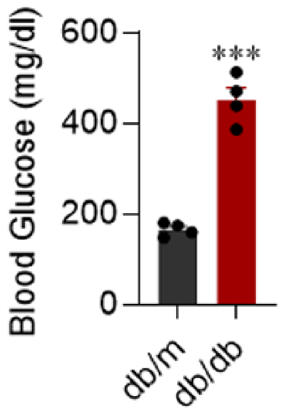

Figure S2. Blood glucose levels in db/db and db/m (control) male mice at age of 8–12 weeks. n=4 per group. ***p < 0.001. Unpaired two-tailed Student's *t* test.

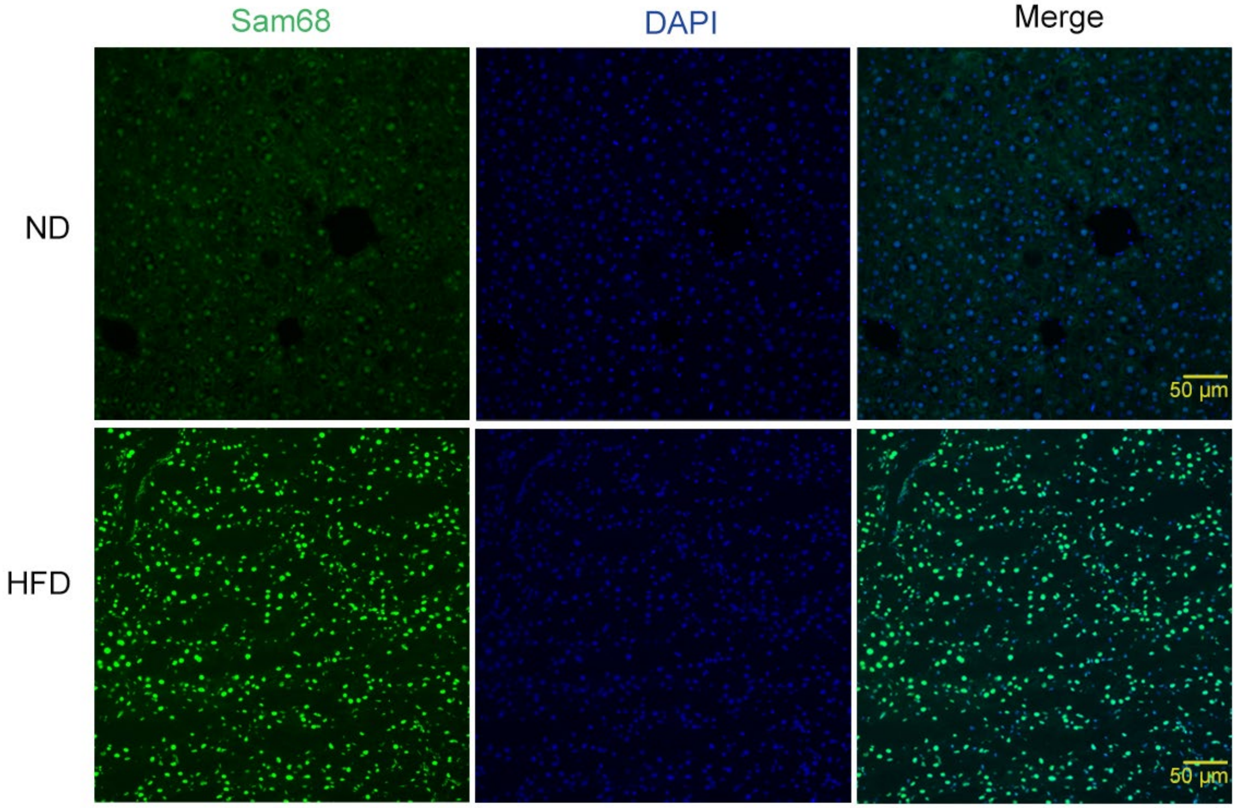

Figure S3. Sam68 protein expression in the nucleus of hepatocytes is increased in the HFD-induced obese (prediabetic) mice. Liver sections were obtained from C57BL/6J mice fed on HFD or ND for 12 weeks and stained with anti-Sam68 antibody (green) and DAPI (blue).

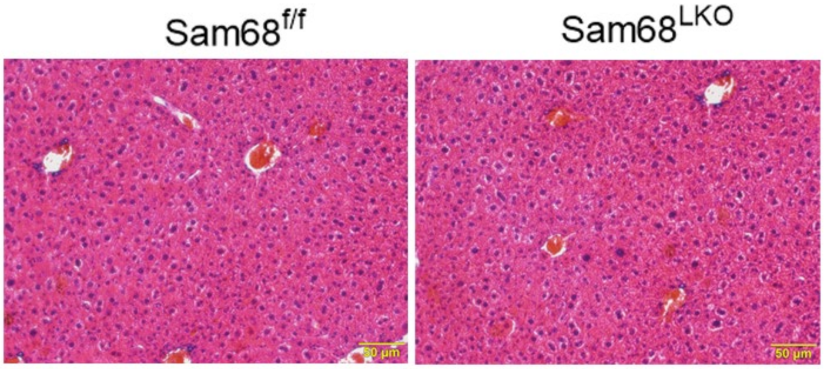

Figure S4. Representative images of H&E staining in the liver of $Sam68^{LKO}$ mice and $Sam68^{f/f}$ mice at 8-12 weeks old.

Supplementary Tables

Table S1. List of PCR primers

	**Forward sequence**	**Reverse sequence**
**For assessment of gene expression with qRT-PCR**		
mSREBP-	GGAGCCATGGATTGCACATT	GGCCCGGGAAGTCACTGT
mPGC-1α	AGCCGTGACCACTGACAACGAG	GCTGCATGGTTCTGAGTGCTAAG
mPEPCK	CCACAGCTGCTGCAGAACA	GAAGGGTCGCATGGCAAA
mG6pase	TGGGCAAAATGGCAAGGA	TCTGCCCCAGGAATCAAAAAT
mβ-actin	GTATGGAATCCTGTGGCATC	AAGCACTTGCGGTGCACGAT
mSam68	GATATCTGTCAGGAGCAGTTTCT	CTCCTCGTCCTCTCACAGATA
hPGC-1α	AACAGCAGCAGAGACAAATGCACC	TGCAGTTCCAGAGAGTTCCACACT
hPEPCK	AAGGAGGATGCCCTGAACCTGAAA	TGCACCTTATGGATGGGAAAGGGA
hG6Pase	TGAATGGCTGCAGTGACCCAGATA	TGGATGTGGAGCCAGTGGAAGAAT
hβ-actin	AGGATGCAGAAGGAGATCACTG	GGGTGTAACGCAACTAAGTCATAG
h-Sam68	GCGAGTGCTGATACCTGTCAAG	TCATTGAGCCCTTTCCCAAT
**For ChIP-qPCR**		
mPGC-1α	GGGCTGCCTTGGAGTGACGTC	AGTCCCCAGTCACATGACAAAG
mG6pase	GGAGGGCAGCCTCTAGCACTGTCA	TCAGTCTGTAGGTCAATCCAGCCC
mPEPCK	GGCCTCCCAACATTCATTAAC	GTAGCCCGCCCTCCTTGCTTTA
**For genotyping of Sam68^{LKO} mice**		
Alb-Cre	TGCCTGCATTACCGGTCGATGC	CCATGAGTGAACGAACCTGGTCG

Table S2. List of antibodies

**Name**	**Catalogue**	**Company Name**
PGC-1α	ab54481	Abcam
G6Pase	ab83690	Abcam
Sam68 for WB and Immunofluorescence	ab76471	Abcam
PEPCK	12940	Cell Signaling
β-actin	8457	Cell Signaling
β-tubulin	2146	Cell Signaling
α-tubulin	2144	Cell Signaling
p-AKT (Thr308)	2965	Cell Signaling
p-AKT (Ser473)	4060	Cell Signaling
Mouse IgG Sepharose Bead Conjugate	3420	Cell Signaling
AKT	9272	Cell Signaling
CRTC2	PA5-72994	Thermo Fisher
Sam68 (7-1) for co-IP	sc-1238	Santa Cruz
Sam68 (7-1) for ChIP	sc-1238AC	Santa Cruz
Anti-mouse IgG, HRP-linked antibody	7076	Cell Signaling
Anti-rabbit IgG, HRP-linked antibody	7074	Cell Signaling
Alexa Fluor 488-conjugated anti-rabbit IgG	A32790	Thermo Fisher
